# Current Status and Issues Associated with Bariatric and Metabolic Surgeries in Japan

**DOI:** 10.1007/s11695-020-05056-4

**Published:** 2020-11-10

**Authors:** Takashi Oshiro, Kazunori Kasama, Taiki Nabekura, Yu Sato, Tomoaki Kitahara, Rie Matsunaga, Motoaki Arai, Kengo Kadoya, Makoto Nagashima, Shinichi Okazumi

**Affiliations:** 1 Department of Surgery, Toho University Sakura Medical Center, 564-1 Shimoshizu, Sakura, Chiba 285-8741 Japan; 2 Weight Loss and Metabolic Surgery Center, Yotsuya Medical Cube, Tokyo, Japan

**Keywords:** Japan, Bariatric and metabolic surgery, Insurance system, Indication, Accreditation and training system, National registry

## Abstract

Among Asian countries, laparotomic and laparoscopic bariatric surgeries were introduced in Japan after its establishment in Taiwan. However, despite high prevalence of potential patients with obesity and diabetes, the wider incorporation of surgery into treatment regimen has been stalling for decades in Japan. While the unique Japanese national health insurance system has guaranteed fair healthcare delivery, it might have worked as a barrier to the development of bariatric and metabolic surgeries (BMS). The present article reviews the status of BMS in Japan and discusses recent issues related to its use. To focus on and identify the major obstacles inhibiting the widespread use of BMS, we have comprehensively covered some major areas including the insurance system, surgical indication, accreditation and training system, original research, and national registry.

## Introduction

Open bariatric surgery was first introduced in Japan by Kawamura et al. in 1982, and the laparotomic restrictive procedure has been covered by national health insurance (NHI) since 1988. Laparoscopic bariatric and metabolic surgeries (BMS) such as Roux-en-Y gastric bypass (LRYGB), adjustable gastric banding (LAGB), and sleeve gastrectomy (LSG) were adopted as self-funded treatment or as part of research projects at limited numbers of hospital by the early 2000s in Japan. LSG was approved as a highly advanced medical treatment (HAMT) since 2010 and was upgraded to full insurance coverage in 2014. LSG with duodenal–jejunal bypass (LSG-DJB) has been acknowledged by the government as a HAMT since 2018. According to a survey by Japan Consortium of Obesity and Metabolic Surgery (JCOMS), 757 cases of laparoscopic BMS were recorded in 2019 [1].

Prevalence of obesity (body mass index [BMI] ≥ 30 kg/m^2^) in Japanese adults is estimated at 4.2%, which is quite low compared to other countries [2]. However, the number of people with type 2 diabetes mellitus (T2DM) is increasing in Japan, with 7.39 million Japanese adults being diabetic [3]. A survey by the Asia-Pacific Metabolic and Bariatric Surgery Society (APMBSS) in 2018 showed that the ratio of number of BMS to obese population in Japan was 0.0103%, which was 5 times less than the average of Asia-Pacific regions (0.0571%) [4].

The present review summarizes the current situation of BMS and focuses on important issues for further development of this field in Japan.

## BMS in Japan

### Current Status of BMS

**Fig. 1:**
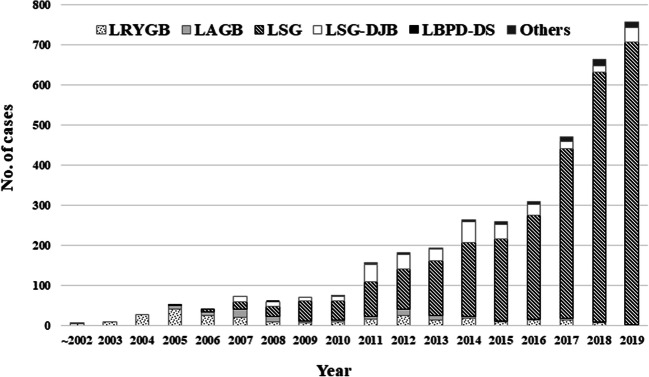
Annual changes in laparoscopic bariatric and metabolic procedures. Adapted from the 2019 JCOMS survey [1]

According to JCOMS survey, 3669 laparoscopic BMS were conducted during 2000 to 2019. The most popular procedure used was LSG (*n* = 2866), followed by LSG-DJB (*n* = 337), LRYGB (*n* = 280), and LAGB (*n* = 109), respectively [1] (Fig. 1). A total of 757 laparoscopic BMS were performed in 2019 at 56 institutions, among which LSG accounted for 93%, LSG-DJB for 4.8%, and LRYGB for 0.2%. LAGB was discontinued in 2019. There was 1 high-volume center (> 100 cases/year). Thirty-nine hospitals performed less than 10 cases/year, and all the procedures conducted were LSG. Up to postoperative 7 years, all procedures resulted in over 50% of excess weight loss (= 100 × (initial weight − postoperative weight)/(initial weight − ideal weight) [ideal BMI of 25 kg/m^2^]). JCOMS survey also indicates that bypass surgery showed superior results as compared to LSG or LAGB in terms of body weight loss (BWL) [1]. Unfortunately, overall postoperative data regarding the impact of these surgeries on obese-related comorbidities was not available in any national resources.

### Safety of BMS

Since the initiation of laparoscopic BMS in Japan, the total rates of morbidity and reoperation were, respectively, 11.8% and 16.7% for LAGB, 29.8% and 11.8% for LRYGB, 16.8% and 2.8% for LSG, and 13.6% and 6.6% for LSG-DJB. Compared to LSG, higher rate of reoperation in LSG-DJB was mainly due to bleeding. In addition, anastomotic-related complications including leakage, stenosis, and marginal ulcers in the earlier days contribute to the higher rate of morbidity and reoperation in LRYGB. With respect to the complications associated with LSG, the rates of postoperative bleeding, leakage, stricture, and gastroesophageal reflux disease (GERD) were 0.7%, 0.5%, 1.1%, and 12.1%, respectively [1].

### Medical Insurance System in Japan

Patients enrolled in the NHI have to pay 30% or less of their total bill. In addition, the patients’ burden is kept below the specified limits under the high-cost medical care benefit system, which compensates for excessive medical costs. Despite the NHI system, a reasonable waiting period for the surgery, because of the free access, is also another advantage. However, under the NHI system, medical practices approved by the Ministry of Health, Labour and Welfare (MHLW) are only allowed. Otherwise, individual must afford the complete medical costs personally.

In 2020, total cost of LSG is approximately $9000 and is covered by the NHI only when performed at MHLW-accredited hospitals. However, with the high-cost medical care benefit system, most patients can undergo LSG for $800 or less.

The HAMT system is another unique framework of medical care in Japan. Newly developed medical technologies should be approved by national council via this verification system in order to be covered by NHI. Patients receiving HAMT at a specially authorized medical facility have to bear only for surgical cost, and other hospital charges are covered by NHI. LSG-DJB is one of the HAMTs since 2018 with an estimated cost of $8000. Procedure is yet not listed to be covered by NHI.

Other BMS including LRYGB, a gold standard and best procedure for severe GERD [5], are not covered by NHI. Moreover, LRYGB is performed in very few patients because self-paid care is available at limited number of hospitals in Japan. The cost of LRYGB ranges from $14,000 to $19,000.

NHI coverage for revisional surgery is not clearly mentioned. Likewise, body contouring surgery following BMS is not subject to NHI coverage.

### Indications for BMS in Japan

In 2020, the indications for LSG and LSG-DJB under the NHI system are shown in Tables 1a and 1b [6, 7], respectively.

**Table 1a:** Indications for LSG in Japan

BMI ≥ 35 kg/m^2^ If there are one or more following obesity-related comorbidities: T2DM, hypertension, dyslipidemia, or OSAS, on the premise that all appropriate non-surgical measures have been tried for at least 6 months before deciding the surgery. BMI between 32.5 and 34.9 kg/m^2^ If there are inadequately controlled T2DM (HbA1c ≥ 8.4% despite medical treatment for at least 6 months) and one or more medically intractable obese-related comorbidities such as: hypertension (systolic blood pressure ≥ 160 mmHg despite antihypertensive medication for at least 6 months), dyslipidemia (LDL cholesterol ≥ 140 mg/dL or non-HDL cholesterol ≥ 170 mg/dL, despite the use of lipid-lowering agents for at least 6 months), or severe OSAS (apnea–hypopnea index ≥ 30).

**Table 1b:** Indications for LSG-DGB in Japan

BMI ≥ 35 kg/m^2^ or above with inadequately controlled T2DM, which refers to the condition of T2DM that requires insulin use or ABCD score* 5 or less.	

*LSG*, laparoscopic sleeve gastrectomy; *LSG-DGB*, LSG with doudenojejunal bypass; *T2DM*, type 2 diabetes; *OSAS*, obstructive sleep apnea syndrome

*ABCD score proposed by Lee et al. [6]

Inadequately controlled T2DM was stipulated based on the results from a multicenter study in Japan [7]

## Issues Associated with BMS in Japan and Proposed Recommendations

### Flaw of the NHI System for Treating Complications

Sporadic postoperative complications such as leakage and stricture or possible complications like GERD have imposed troubles for surgeons. This is because, due to regulatory obstacles, there are limitations in using efficacious devices for the management of such complications. Off-label use is strictly prohibited unless authorized by the MHLW.

## Recommendations: Loosen Regulations on Necessary Devices

Standard endoscopical treatment tools such as over-the-scope clip, retrievable stent, or achalasia balloon have already received Japan’s pharmaceutical affairs regulatory approval for other purposes. For widespread adoption of BMS, creating safe financial and flexible regulatory environment is needed not only for patients but also for surgeons.

### Barriers to Widespread LSG-DJB Adoption

It may be no exaggeration to say that LSG is virtually the only option of BMS for patients with obesity in Japan. However, LSG seems to be less effective in BWL for super-obesity, as observed even in short-term follow-up [8]. In addition, patients with severe T2DM were unlikely to achieve diabetes remission after LSG [7]. LSG-DJB could be one of the optional procedures to overcome these problems. LSG-DJB is a combination of LSG and proximal intestinal bypass, in which exploration of the remnant stomach can be performed easily by standard endoscopy unlike LRYGB [9].

According to two multicentric studies in Japan [7, 10], nearly half of the patients with obesity and T2DM have ABCD score ≤ 5, which meets the indication criteria of LSG-DJB. However, the number of LSG-DJB was limited to only 12 cases at 2 accredited hospitals in 2019. High surgical cost and strict accreditation criteria for a BMS center to conduct LSG-DJB as HAMT have hindered the acceptance of LSG-DJB. The surgeon needs experience of handling 7 cases of LSG-DJB in advance; moreover, these cases must be done with self-paid and/or hospital fund as per the current NHI rules. Until now, only two hospitals have been approved as accredited hospitals for LSG-DJB. Meanwhile, nearly 300 cases of LSG-DJB have been carried out with self-paid care at the high-volume center (Yotsuya Medical Cube, Tokyo), where LSG-DJB was introduced by Kasama in 2007 [11], but it has not been approved as an accredited institution due to the lack of intensive care unit.

## Recommendations: Ease the Restrictions on LSG-DJB Use

Considering their contribution to the development of LSG-DJB, the pioneer and experienced hospital should be immediately approved as accredited institution. We have already demonstrated short- and mid-term outcomes regarding well-balanced safety and efficacy of LSG-DJB in Japanese population [7, 9, 10]. It is the appropriate time that LSG-DJB should be fully covered by NHI and commonly provided at authorized institutions.

### Strict Indication of BMS for Patients with Lower BMI Obesity and T2DM

Saiki et al. indicated that the number of individuals having T2DM with obesity of BMI ≥ 35 kg/m^2^ is estimated to be at least 256,000, who meet the indication criteria of LSG in Japan [12]. Considering that the Japanese population tends to develop T2DM with lower BMI obesity, there are more potential candidates for BMS in Japan [13]. In response to the 2nd Diabetes Surgery Summit (DSS-II) recommendations [14], the government approved the indication expansion for lowering BMI in 2020 (Table 1a). However, compared to any other guidelines ever published worldwide, Japan’s indication criteria of BMS for patients with lower BMI obesity are stringent and complicated without credible scientific rationale. It would be extremely difficult for physicians to find eligible candidates for the surgery. In fact, only one case met this strict indication criteria among individuals who underwent BMS (1500 cases) at Yotsuya Medical Cube in the past 14 years.

## Recommendations: Amend Indication for Patients with Lower BMI Obesity

The surgery should be available as an option to use when appropriate and not only when all other options have been eliminated. BMS might serve as a potential opportunity for preventing co-morbid diseases and complications of obesity. The indication of BMS for patients with lower BMI obesity should be revised in effective ways in line with the original intention of DSS-II guideline.

### Obesity with GERD

Most Japanese surgeons currently consider the presence of severe reflux esophagitis (RE) a contraindication to LSG. Studies in other countries have indicated that crural repair at the time of LSG could prevent de novo or worsening of reflux symptoms after the surgery [15]. However, additional concomitant crural repair during LSG is only performed at a limited number of hospitals in Japan because of controversial efficacy and non-coverage of repairing procedure by NHI.

## Recommendations: Adopt LRYGB for the Patients with Severe GERD

The potential risk of delayed detection of gastric cancer in bypassed stomach after LRYGB might be concerned. However, the prevalence of *Helicobacter pylori* infection is gradually decreasing in Japan, especially in those aged under 40 years [16]. Furthermore, preoperative screening endoscopy is routinely performed in patients undergoing BMS in Japan. LRYGB should be adopted for eligible patients with severe GERD under NHI coverage if they have only minimum risk of gastric cancer.

### Restrictive BMS Options

Bypass surgeries such as LRYGB and LSG-DJB may be an adequate procedure to accomplish better diabetes remission in Japan [7, 10]. Similarly, LSG for super-obesity seems to be less efficient in BWL compared to bypass surgery [8].

In long-term follow-up, insufficient weight loss after primary LSG might necessitate further surgical interventions [17]. In Japan, re-sleeve gastrectomy may be a realistic surgical option for weight loss failure in patients with primary or secondary dilation of gastric tube at the moment. Except for two-staged LSG-DJB, conversion from LSG to bypass surgery is not supported by NHI coverage.

## Recommendations: Broaden Available BMS Options

Some Asian countries adopted NHI coverage for BMS with region variation in operation type including bypass surgery. Although the number of BMS in the Asian region has increased rapidly, the frequency of BMS per obese population in Japan is the lowest among these countries (Table 2). Thus, we need variety of BMS, including bypass surgery, along with public health insurance coverage for achieving better T2DM resolution and long-term weight loss.

**Table 2:** Indication of bariatric surgery, type of procedures, annual number of surgeries, and frequency of surgery per obese population

Country	Indication of bariatric surgery (BMI)	Type of surgery	Annual procedures in 2017	F/OP
Japan	≥ 35 with disease	SG, SG-DJB	471	0.0103%
Korea	≥ 35 or ≥ 30 with disease	SG, RYGB, OAGB, BPD-DS, GP, AGB	438*	0.0225%
Taiwan	≥ 37 or ≥ 32 with disease	SG, RYGB, OAGB, SG-DJB	2834	0.1803%
Singapore	≥ 37.5 or ≥ 32.5 with disease	SG, RYGB	428	0.1548%
India	≥ 37.5 or ≥ 32.5 with diseases	SG, RYGB, OAGB, BPD-DS, AGB	14,543	0.0436%

Adapted from Ohta and Angrisani et al. [4, 18]

*SG*, sleeve gastrectomy; *SG-DJB*, sleeve gastrectomy with duodenojejunal bypass; *RYGB*, Roux-en-Y gastric bypass; *OAGB*, one-anastomosis gastric bypass; *BPD-DS*, biliopancreatic diversion with duodenal switch; *GP*, gastric plication; *AGB*, adjustable gastric banding; *F/OP*, frequency of surgery per obese population

*The data of 2016 was used in Korea

Based on scientific evidence, the national health insurance service (NHIS) drastically extended its national insurance coverage to patients with obesity problems since January 2019 in Korea. The indications for bariatric surgery and applicable laparoscopic operations are shown in Table 2. Metabolic surgery was adopted to control T2DM. LRYGB and LSG are recommended for patients with BMI ≥ 27.5 kg/m^2^ and medically uncontrolled T2DM. Reversal and revisional surgeries are covered by NHIS. All medical practices related to perioperative management are also covered by NHIS, and the percentage of reimbursement for surgical cost depends on the degree of BMI [19]. Taking long-term cost savings and effectiveness into consideration, the BMS programs in Korea might be useful as a reference for modifying/amending the Japanese program.

### Accredited System and Training System for BMS

Most cases of BMS in Japan so far have been performed by qualified experienced laparoscopic surgeons [20] at a limited number of and specially accredited medical facilities. In principle, accredited centers for BMS approved by the government need to be equipped with multidisciplinary team and hospital equipment to meet institutional requirements in accordance with the guidelines issued by the Japanese Society for the Surgery of Obesity and Metabolic Disorders (JSSO) [21].

Unfortunately, most of the surgeons continue to perform only LSG after institutional accreditation, and an optimal training system has not yet been established in Japan. Japanese surgeons might, therefore, miss the opportunity to learn fundamental skills such as lifetime follow-up care, management of complications, other standard BMS, and revisional surgery.

For more than a decade, only one high-volume private hospital has provided a 1- or 2-year fellowship program for doctors from Japan as well as from overseas. The comprehensive course comprises of a balance of operative skills, diagnosis and management of surgical complications, postoperative follow-up, and clinical research. Although the efforts of enthusiastic and experienced experts are greatly appreciated, a few available positions and limited type of procedures present realistic challenges.

## Recommendations: Promote International Collaboration and Cooperation

Training system has now become the core theme in APMBSS, and international fellowship programs for BMS are successfully conducted by some APMBSS member countries. The key to develop a useful training system is to promote international collaboration and cooperation through sharing advantages and benefits of individual countries, especially in Asia.

### Research

Clinical articles related to laparoscopic BMS that were published from Japan were searched using PubMed with the following keywords: obesity, bariatric, metabolic, surgery, and Japan. Only 60 research articles with research work conducted on human subjects could be found. The total number of retrospective and prospective articles was 50 (83%) and 10 (17%), respectively. Twenty-three papers (38%) originated from single high-volume center in Japan. In reference to evidence rating form [22], the number of weak evidence level articles was 46 (77%), whereas there was no strong evidence level report. Now, the first RCT from Japan, titled “Bariatric surgery vs. intensive medical therapy in mildly obese Japanese patients with early onset type 2 diabetes mellitus,” is under preparation.

## Recommendations: Forward-Thinking Approach

Keeping in view the diabetic burden in Japan, there is a tremendous potential for clinical trials and studies from various clinical fields in reference to BMS in the country. However, the lack of funding, lack of trained research personnel, and strict regulatory and ethical approval frameworks for emerging surgical options are significant stumbling blocks that prevent high-quality research to be carried out. There is a need for a more pro-active approach to obtain more realistic and conducive evidence for the development of BMS within and outside Japan.

### National Registry

Domestic registry of JSSO was started in 2012, and JSSO has participated in the IFSO global registry project after the third worldwide survey in 2017. In addition, JSSO also joined the National Clinical Database (NCD) in collaboration with the Japanese Society of Gastroenterological Surgery (JSGS). The NCD is linked to the board certification system of some surgical societies including JSGS. Most surgical cases (≥ 95%) performed in Japan are included in the NCD [23]. In both the JSSO and NCD registry, the data can be registered via a web-based data management system. At present, collected data are mainly focused on pre- and perioperative clinical variables, which include baseline demographic characteristics and anthropometrics data, medical antecedents, comorbidities, operation type, and operative complications and mortality. This means that the feedback data summarize the present state of BMS in Japan and will aid in risk evaluation, considering morbidity and mortality rates in each procedure. Biennial questionnaire survey through postal mail or e-mail was conducted by JCOMS, which might compensate for the lack of follow-up data of BMS in Japan. Although information on postoperative body weight changes over time was collected, queries do not include the outcomes of comorbidities.

## Recommendations: Modify Registration System

The fundamental problems of the current registration system in Japan are lack of mid- and long-term follow-up data, data collection by standardized outcomes reporting, and real-time analysis and feedback to healthcare providers. By resolving these issues, national registry data could be used to evaluate the safety and efficacy of BMS in a standardized fashion. It could also help in establishing the best practices for treating obesity and comorbidities in Japanese citizen and influence national health policies in an appropriate manner. Considering that not many BMS are performed in Japan at present, we have a golden opportunity and less complications to revise the platform of registration system before the issues become more complex and difficult to be fixed.

## Conclusion

Japanese citizens holding proof of insurance have the opportunity to avail the benefits of the universal health coverage system for BMS. However, strict regulations of this system could be a bottleneck for free access to mandatory devices for treating operative complications and adoption of diverse surgical options available.

Despite consistently supportive evidence of clinical effectiveness and socioeconomic advantages of BMS, acceptance of surgeries by the general public and physicians in Japan lags far behind the recommendations by DSS-II and other related societies. To provide sustainable high-standard care to all the patients, updating reimbursement policies based on the collection and analysis of data from standardized outcomes reported around Japan is needed. The keys to achieve the goal of better and wider use of various types of BMS as per requirement of the individual are proper perspective in terms of a better training system, more focus on research, commitment to the national registry, more governmental support, and broad insurance coverage of BMS.
